# Ancient Divergence in the Trans-Oceanic Deep-Sea Shark *Centroscymnus crepidater*

**DOI:** 10.1371/journal.pone.0049196

**Published:** 2012-11-08

**Authors:** Regina L. Cunha, Ilaria Coscia, Celine Madeira, Stefano Mariani, Sergio Stefanni, Rita Castilho

**Affiliations:** 1 CCMAR-CIMAR, Universidade do Algarve, Faro, Portugal; 2 Institute of Biological, Environmental and Rural Sciences (IBERS), Aberystwyth University, Aberystwyth, Ceredigion, United Kingdom; 3 School of Environment and Life Sciences, University of Salford, Manchester, United Kingdom; 4 IMAR / DOP, Department of Oceanography and Fisheries, University of the Azores, Horta, Azores, Portugal; University of York, United Kingdom

## Abstract

Unravelling the genetic structure and phylogeographic patterns of deep-sea sharks is particularly challenging given the inherent difficulty in obtaining samples. The deep-sea shark *Centroscymnus crepidater* is a medium-sized benthopelagic species that exhibits a circumglobal distribution occurring both in the Atlantic and Indo-Pacific Oceans. Contrary to the wealth of phylogeographic studies focused on coastal sharks, the genetic structure of bathyal species remains largely unexplored. We used a fragment of the mitochondrial DNA control region, and microsatellite data, to examine genetic structure in *C. crepidater* collected from the Atlantic Ocean, Tasman Sea, and southern Pacific Ocean (Chatham Rise). Two deeply divergent (3.1%) mtDNA clades were recovered, with one clade including both Atlantic and Pacific specimens, and the other composed of Atlantic samples with a single specimen from the Pacific (Chatham Rise). Bayesian analyses estimated this splitting in the Miocene at about 15 million years ago. The ancestral *C. crepidater* lineage was probably widely distributed in the Atlantic and Indo-Pacific Oceans. The oceanic cooling observed during the Miocene due to an Antarctic glaciation and the Tethys closure caused changes in environmental conditions that presumably restricted gene flow between basins. Fluctuations in food resources in the Southern Ocean might have promoted the dispersal of *C. crepidater* throughout the northern Atlantic where habitat conditions were more suitable during the Miocene. The significant genetic structure revealed by microsatellite data suggests the existence of present-day barriers to gene flow between the Atlantic and Pacific populations most likely due to the influence of the Agulhas Current retroflection on prey movements.

## Introduction

The paucity of data on deep-sea sharks due to the intrinsic difficulty in obtaining samples, have hampered our understanding of the genetic structure of the species inhabiting these remote environments. Nevertheless, a number of expectations based on the prior knowledge of this group of species can be formulated. Dispersal in elasmobranchs is mostly determined by the swimming ability of juveniles and adults, as they have no pelagic larvae. Vagility increases with body size and tends to be higher in pelagic or benthopelagic oceanic species, whereas benthic and coastal species often exhibit low dispersal abilities [1].

Large oceanic elasmobranchs are expected to exhibit low levels of genetic differentiation across vast stretches of the open ocean whereas smaller benthic species may display stronger genetic differentiation. While there is a relatively large number of phylogeographic studies focused on coastal sharks [2], [3], [4], [5], the patterns of genetic structure of deep-sea species remain virtually unknown (but see [6] for a recent study on the population structure of a deep-water squaloid shark).

An increasing number of studies in elasmobranchs reported genetic discontinuities within ocean basins, revealing the existence of barriers to gene flow [7], [8]. Most of these genetic breaks were shaped by female philopatry (return to natal sites to breed [9], [10], [11]) or vicariant events [12].

Paleoceanographic changes including the opening and closure of corridors connecting marine basins triggered major climate fluctuations as well as faunal turnover. There is increasing evidence that Tethys final closure at 14 myr [13], [14] promoted splitting events between Atlantic and Indo-Pacific lineages in several groups of elasmobranchs as described in scalloped hammerhead sharks [15] or in angel sharks [12].

The long-nosed velvet dogfish *Centroscymnus crepidater* (Barbosa du Bocage & de Brito Capello, 1864) is a deep-sea shark with an estimated age of 20 years for female maturity [16]. It occurs in depths ranging from 650 to 1650 m (bathyal) [1] and is thought to be unlikely to undertake inter-oceanic migrations via long-range active dispersal, in the way that oceanic pelagic species do. Nevertheless, *C. crepidater* exhibits a worldwide distribution occurring on the continental and insular slopes of the eastern Atlantic (Iceland to southern Africa), Indian Ocean (Aldabra Islands and India) and Pacific (northern Chile, New Zealand, and southern Australia) [17]. There is no information regarding migration routes that could shed some light on contemporary levels of gene flow; thus, it is difficult to speculate as to whether its broad distribution is a remnant of ancient vicariance or is sustained by some degree of recent gene flow, possibly following a stepping-stone model [18].

*C. crepidater* belongs to the family Somniosidae, which the paleontological record dates back to the Campanian [83.5−70.6 myr] [19], [20]. The only fossils shark teeth unambiguously assigned to the genus *Centroscymnus* are from the Iseyama Formation, central Japan [21] of the Middle Miocene [16.4−11.2 myr]. Given that the fossil record of the genus *Centroscymnus* dates back to the Middle Miocene, and considering its broad distribution with populations occurring both in the Atlantic and Indo-Pacific basins, this tectonic event might have shaped the genetic structure of this species.

Here, we used an 868 bp-fragment of the mitochondrial DNA (mtDNA) control region (CR) to analyse genetic structure in *C. crepidater* collected in the Atlantic and southern Pacific Oceans. We further explore phylogenetic patterns using a portion of the mtDNA cytochrome oxidase subunit I (COI) gene. Genetic diversity and connectivity between Atlantic and Pacific populations was examined with seven microsatellite loci. The combined use of mtDNA and microsatellite data allows inferring past evolutionary events [22] as well as present-day gene flow [23], [24]. Mitochondrial data were also used to determine if genetic structure in *C. crepidater* reflects any female philopatric behaviour, and to explore if the Tethys closure triggered lineage-splitting events within this species. Given that mtDNA is maternally inherited, it would allow assessing female philopatry. As a result of the Tethys vicariant event, the predicted phylogenetic pattern would be a sister-clade relationship between lineages from the eastern Atlantic and Indo-West Pacific basins [25], [26].

## Results

### Genetic Diversity and Population Structure of *C. crepidater*

#### Mitochondrial data

**Figure 1:**
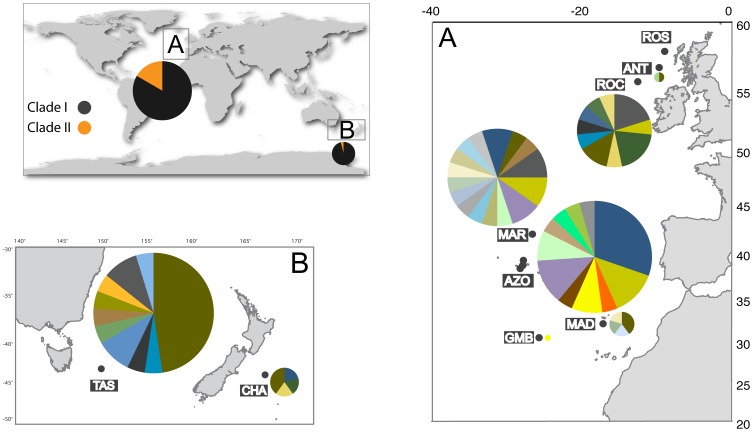
Sampling locations of *Centroscymnus crepidater*. Haplotype frequencies and clade proportions (Clade I, in black and Clade II, in orange) based on the CR mtDNA data set are also depicted. The size of each slice is proportional to the number of individuals sharing the same haplotype. The letters A and B refer to the Atlantic and Pacific sampling locations, respectively. Sampling location codes: ROS (Rosemary Bank); ANT (Anton Dohrn Seamount); ROC (Rockall Trough); MAR (Mid-Atlantic Ridge); AZO (Azores); MAD (Madeira); GMB (Great Meteor Bank); TAS (Tasman Sea), and CHA (Chatham Rise).

Sequencing 868 bp of the mtDNA CR from 92 individuals (GenBank accession numbers: JQ360863-JQ360954) of *C. crepidater* (Atlantic: 66 specimens; Pacific: 26 specimens) yielded a total of 70 variable sites and 45 haplotypes (Fig. 1). Additionally, the 655-bp fragment of the cytochrome oxidase subunit I (COI) mtDNA gene yielded total of 61 variable and 11 parsimony-informative sites. We used two mtDNA markers to assess if the reconstructed phylogenetic patterns were congruent. All phylogenetic analyses were performed separately for each gene. The CR (-ln L  =  −2407.44) and COI (-ln L  = 1239.12) ML trees showed two deep-divergent clades (Material S1 and S2, respectively).

**Figure 2:**
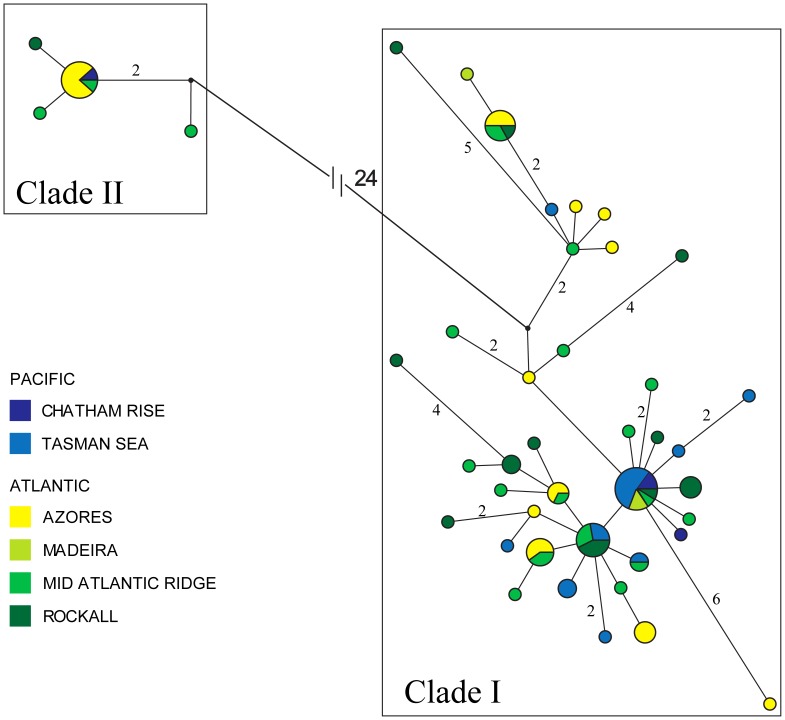
Median-joining network of 45 mitochondrial control region haplotypes of *Centroscymnus crepidater*. The area of each circle is proportional to the number of individuals sharing a particular haplotype. Colors refer to sample location, and the size of each slice is proportional to the number of individuals with that haplotype. Haplotypes are connected by branch lengths approximately equal to the inferred mutational steps. Numbers represent mutational steps.

**Figure 3:**
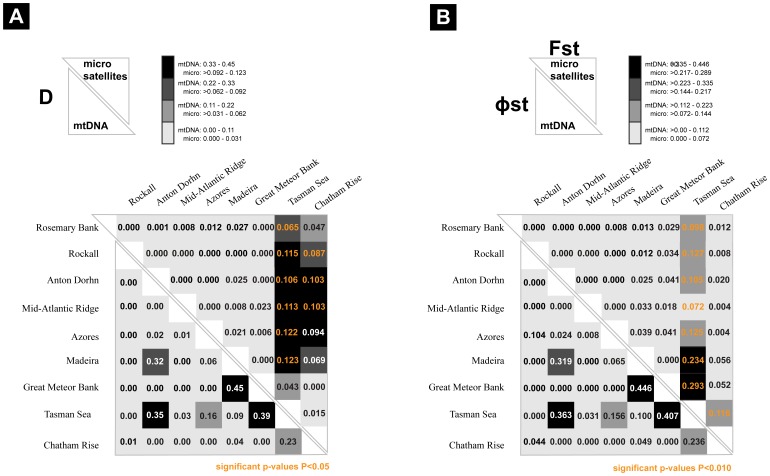
Measures of genetic differentiation between geographic locations. (**A**) Location pairwise D values for the mitochondrial (below diagonal) and microsatellite (above diagonal) data sets. (**B**) Location pairwise Φst for the mitochondrial (below diagonal) and F_st_ for the microsatellite (above diagonal). The scale on the top of each figure indicates the correspondence between class intervals and colored boxes. Significant values after correction are depicted in orange.

The haplotype network based on CR sequence data also shows two well-defined clades (hereafter clades I and II) separated by 24 mutational steps (Fig. 2). Clade I included a larger number of diverse individuals (N = 80; 41 haplotypes, *h* = 0.886±0.029 and π = 0.0037±0.00038 (Table 1). Clade II with fewer individuals (N = 12; 4 haplotypes) showed lower haplotype and nucleotide diversities (*h* = 0.455±0.170, π = 0.00098±0.00049). Corrected net sequence divergence between clades I and II is 3.1%±0.61%, with an average sequence divergence (overall mean) between individuals of 1.0%±0.17%. The pairwise estimates of *D* were only significant between the Azores and the Tasman Sea. Between sites, no pairwise Φst were significant (Fig. 3). The individuals from each clade are equally distributed between ocean basins (*p* = 0.168, two-tailed Fisher’s exact test).

**Table 1:** Genetic diversity for all *Centroscymnus crepidater* sampled locations.

Location	Acronym	Mitochondrial DNA				Microsatellites						
		N	NH	h (± s.d.)	π (± s.d.)	N	A	A_R_	H_O_	H_E_	G_IS_	
Rosemary Bank	ROS	0	–	–	–	26	4.9	3.2	0.522	0.481	−0.085	*
Anton Dohrn Bank	DOR	2	2	1.000 (±0.500)	0.008 (±0.009)	16	4.4	3.4	0.554	0.516	−0.073	
Rockall Trough	ROC	15	10	0.933 (±0.045)	0.009 (±0.005)	25	4.4	3.2	0.583	0.491	−**0.187**	***
Mid-Atlantic Ridge	MAR	20	17	0.984 (±0.021)	0.013 (±0.007)	35	5.7	3.3	0.522	0.495	−0.055	
Azores	AZO	23	11	0.885 (±0.048)	0.018 (±0.009)	20	4.3	3.3	0.491	0.529	0.072	
Madeira	MAD	5	4	0.900 (±0.161)	0.003 (±0.002)	7	3.4	3.4	0.524	0.531	0.013	
Great Meteor Bank	GMB	1	1	0.000 (±0.000)	0.000 (±0.000)	3	3.2	–	0.533	0.667	0.200	
Tasman Sea	TAS	21	8	0.624 (±0.121)	0.002 (±0.001)	22	3.3	2.4	0.227	0.303	0.251	
Chatham Rise	CHA	5	4	0.900 (±0.161)	0.014 (±0.017)	6	2.9	2.9	0.381	0.426	0.106	
All	–	92	45	0.946 (±0.013)	0.011 (±0.006)	160	7.4	3.3	0.481	0.485	0.009	*

For mitochondrial CR sequence data: number of individuals (N), number of haplotypes (NH), haplotype diversity (*h*), nucleotide diversity (π), and standard deviation (s. d.). For microsatellite data: number of individuals (N), mean number of alleles across loci (A), allelic richness (A_R_), observed (H_O_) and expected (H_E_) heterozygosities and heterozygote deficiency (G_IS_). Significance levels are indicated (*p<0.05, ***p<0.001); values in bold indicate significance after q-value correction).

Results of AMOVA between the two oceans (Atlantic and Pacific) using mtDNA sequence data indicate absence of genetic structure, with most of the variation allocated within sites (Table 2).

**Table 2:** *Centroscymnus crepidater*.

Marker	Group composition	% variation	Fixation Index		*P*
mtDNA	Atlantic *vs* Pacific				
	Between groups	−1.12	Φ CT =	−0.011	0.510
	Among locations within groups	3.81	Φ SC =	0.038	0.281
	Within locations	97.31	Φ ST =	0.027	0.238
	Within Atlantic (one gene pool)				
	Among locations	1.19			
	Within locations	98.81	Φ ST =	0.010	0.348
	Within Pacific (one gene pool)				
	Among locations	22.93			
	Within locations	77.07	Φ ST =	0.229	0.129
Microsatellites	Between Atlantic and Pacific				
	Between groups	5.9	FCT =	0.059	**0.000**
	Among locations within groups	0.66	FSC =	0.007	**0.011**
	Within locations	93.44	FST =	0.066	**0.029**
	Within Atlantic (one gene pool)				
	Among locations	0.07			
	Within locations	99.93	FST =	0.001	0.119
	Within Atlantic (2 gene pools): North (ROS, ANT, ROC) *vs* South (MAR, AZO, MAD, GMB)				
	Between groups	−0.19	FCT =	−0.002	0.573
	Among locations within groups	0.19	FSC =	0.002	0.121
	Within locations	100.00	FST =	0.000	0.113
	Within Pacific (one gene pool)				
	Among locations	11.62			
	Within locations	88.38	FST =	0.116	**0.001**

Analysis of molecular variance (AMOVA) of mtDNA control region sequence data and microsatellite data between and within ocean basins. Significant values in bold (see Table 1 for details on sampling locations).

**Figure 4:**
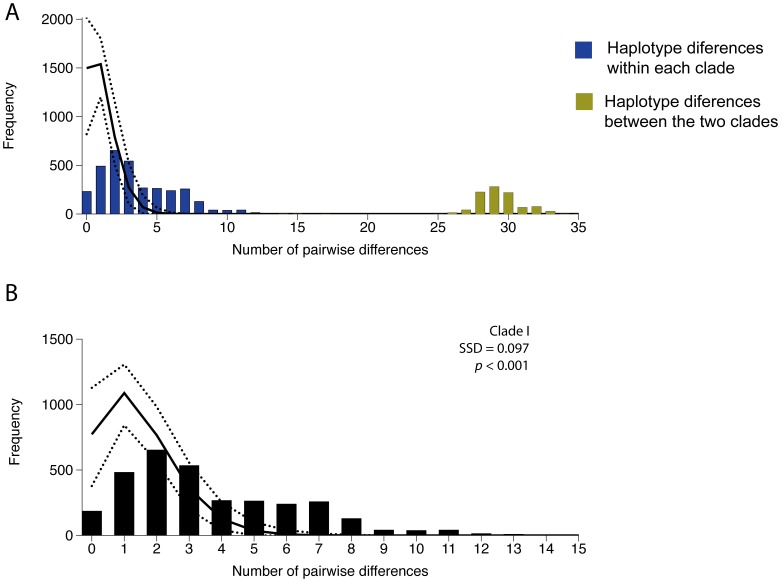
Pairwise mismatch distributions using the CR mtDNA data set. (A) mismatch distribution including all sequences; (B) mismatch distribution including sequences from Clade I only. Vertical bars represent observed frequencies. Upper and lower dashed lines represent the upper and lower 95% limits based on 10000 replicates in Arlequin. Solid line represents the model (expected) frequency.

Mismatch analysis for all the CR mtDNA sequences displays a bimodal distribution with two well-separated peaks (Fig. 4A). The first peak (closer to the origin) represents variation among individuals of each clade whereas the second, with greater differences among haplotypes, represents comparisons between the two. The non-unimodal distribution of pairwise nucleotide differences (Fig. 4B) suggests that clade I is in equilibrium, and a sudden-population-expansion model can be rejected (SSD = 0.097; *P*<0.001).

#### Microsatellite data

A total of 160 individuals of *C. crepidater* from seven Atlantic and two Pacific locations were genotyped for seven microsatellite loci. Gene diversity varied between 0.233 to 0.829 (mean = 0.485) and the total number of alleles per locus varied between 3 to 11 (mean = 7.4) (Table 3). The plots of the distribution of allele frequencies did not show any striking difference within Atlantic locations but showed a few low frequency different alleles between Atlantic and Pacific locations (Material S3). According to Lositan, only locus Ccrep13 was a candidate for balancing selection. Deviation from Hardy–Weinberg equilibrium was found at six locus-by-population combinations out of a total of 49 combinations. There was a heterozygote excess for locus Ccre34 for MAR, ROC, ROS, AZO and a heterozygote deficit in locus Ccre02 for AZO and CHA (see Table 1 for sampling site acronyms). When all loci were taken into account, overall deviation was equivocal, with statistically significant Gis after correction (*P*<0.001) and non-significant Fis (*P*>0.132). If only neutral loci were taken into account both values were not significant after correction (Gis, *P*>1.000, Fis, *P*>0.118).

**Table 3:** Summary statistics for seven microsatellite loci of Atlantic and Pacific samples of *Centroscymnus crepidater:* number of allele counts (N_C_), mean number of alleles across locations (N_A_), mean allelic richness standardized to sample size N = 6, excluding Great Meteor Bank sample (A_R_), effective number of alleles (E_NA_), observed heterozygosity (H_O_), heterozygosity within populations (H_S_), corrected total heterozigosity (H’_T_), and fixation index (Gis).

Locus	N_C_	N_A_	A_r_ (N = 6)	E_NA_	H_O_	H_S_	H’_T_	G_IS_
Ccre2	266	9	5.1	3.2	0.661	0.774	0.788	0.146
Ccre11	316	5	2.5	1.4	0.319	0.275	0.286	−0.159
Ccre12	320	8	4.0	2.2	0.463	0.586	0.631	0.210
Ccre13	310	9	5.1	4.6	0.715	0.829	0.819	0.138
Ccre23	266	7	2.2	1.3	0.133	0.233	0.212	0.429
Ccre27	320	11	2.1	1.3	0.258	0.253	0.273	−0.021
Ccre34	314	3	2.1	1.8	0.816	0.446	0.493	−0.830
Overall	NA	7.4	3.3	2.2	0.481	0.485	0.500	0.009

(NA, not applicable).

**Figure 5:**
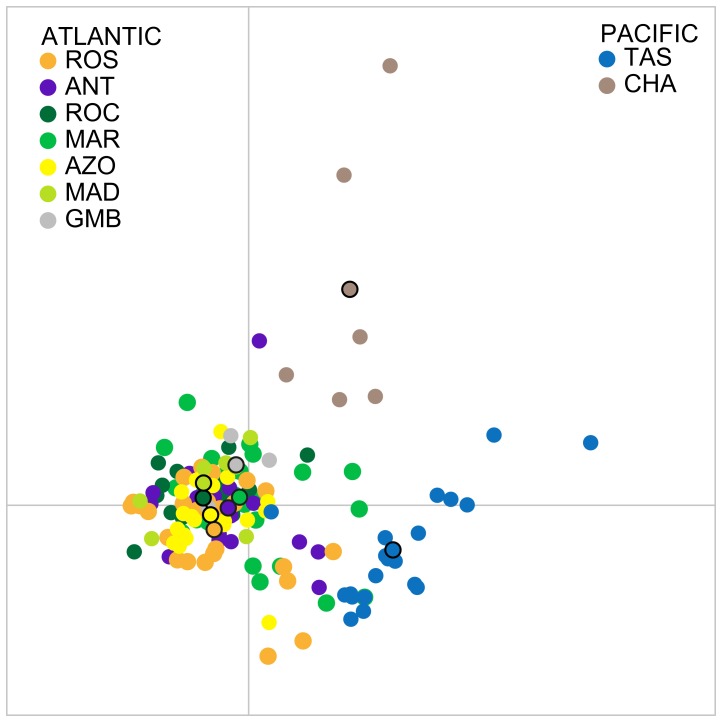
Discriminant analysis of principle components (DAPC) of multi-locus genotype data for all populations. Individual genotypes appear as circles, and black stroke circles represent the center of dispersion of each group. Populations are depicted in colors. X and Y axes are the first two principle components.

The analytical approaches to detect genetic structure were congruent in returning absence of structure within the Atlantic samples but significant differentiation between Atlantic and Pacific. Visual inspection of the scatterplots of the first two principal components of discriminant analysis (representing 29.4% and 8.9%) (Fig. 5) showed that the first axis separates mostly the Atlantic from the Pacific. Atlantic samples form a tighter cluster than the Pacific samples, with the second axis slightly setting apart the Chatham Rise. The percentage of Atlantic individuals correctly assigned to their location of origin was lower than 46%. Pacific individuals were assigned 67% (Chatham Rise) and 91% (Tasman Sea) to the location of origin.

Results from Bayesass showed that contemporary migration between ocean basins is occasional and the direction is predominantly from the Pacific, with 3.8% of individuals migrating into the Atlantic, whereas only 1.9% of the migration occurs in the opposite direction.

Geneland analyses recovered three major clusters corresponding to the following locations: (1) Pacific, (2) Atlantic, and (3) Great Meteor Bank (Material S4A). Great Meteor Bank has only 3 scored individuals with high proportion of missing data. Removing the individuals from this location from the dataset, only two clades were recovered corresponding to the Pacific and Atlantic locations (Material S4B).

### Divergence Time Estimates in *C. crepidater*

**Figure 6:**
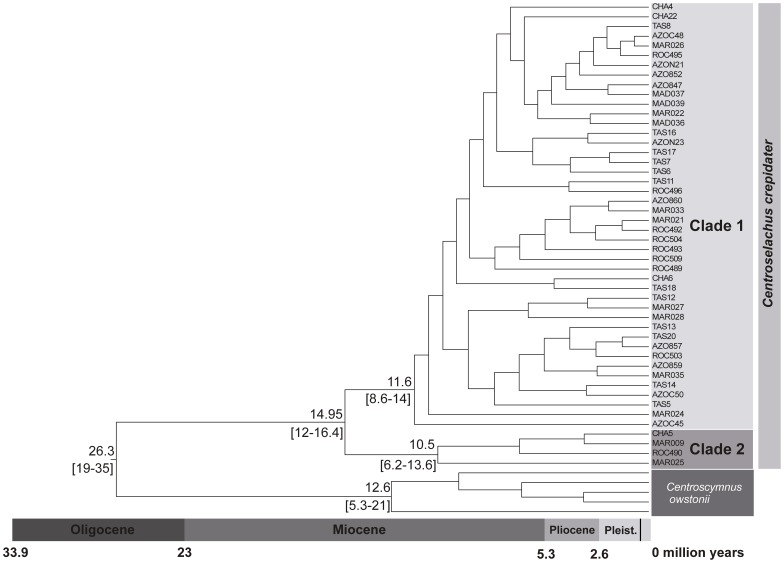
Bayesian dating analysis based on the CR mtDNA data set obtained with Multidivtime. Age estimates (in million years) of the main lineage splitting events within *Centroscymnus crepidater* are depicted. Values in square brackets represent the 95% confidence intervals. Sample codes: ROC (Rockall Trough); MAR (Mid-Atlantic Ridge); AZO (Azores); MAD (Madeira); TAS (Tasman Sea), and CHA (Chatham Rise).

Multidivtime dating analysis based on the mitochondrial CR data set estimated the divergence between the two main clades at 14.95 [12–16.4] myr ago (Fig. 6). The age of the most recent common ancestor of clade I was estimated at 11.6 [8.6–14] myr, and of clade II at 10.5 [6.2–13.6] myr. The split between *C. crepidater* and *Centroscymnus owstonii* was estimated at about 26 myr ago.

## Discussion

Phylogenetic analyses based on mtDNA recovered two deeply divergent clades (24 mutations apart) with an origin dating back to the Miocene. These genetic clades have different abundances (Clade I = 80 individuals, 87%; Clade II = 12 individuals, 13%) and are equally distributed between ocean basins (non-significant *p*-value of the two-tailed Fisher’s exact test) without a discernible geographic pattern. In contrast, microsatellite data indicated a strong genetic differentiation between the Atlantic and Pacific Oceans.

Two main caveats must be addressed prior to interpreting these results. Data from the Pacific rely on fewer individuals from two locations and therefore we have a restricted representation of the genetic diversity of the region. We also have a limited number of microsatellite loci. Thirteen pairs of primers were initially developed for this species [27]. However, we were limited to use seven loci, as the remaining six pairs were monomorphic. In any case, the genetic assessment of a benthopelagic shark species is a contribution in itself, as genetic architecture of sharks, especially deep-water species, is notoriously less studied than their marine teleost counterpart.

### Taxonomic Status and Genetic Architecture of *C. crepidater*

The observed deep split of 24 mutations between the two main CR mtDNA clades (Fig. 2) suggests the existence of two different taxonomic entities. However, there is compelling evidence to conclude that specimens from both clades belong to the same species. First, sequence divergence between clades I and II (3.1%±0.61%) is much smaller when compared to the closely related species *Centroscymnus owstonii* and *Centroscymnus coelolepis*, used here as outgroups (17.9% and 18%, respectively). Second, the level of interspecific divergence in sharks is generally higher than in bony fishes (e.g. in six scalloped hammerhead shark species, sequence divergence ranged between 7.8 and 24.3% [15], and in seven angel sharks species ranged from 4.9 to 9.4% [12]). Finally, the overall mitochondrial sequence divergence in *C. crepidater* (1.0%*)* is similar to the value found in other shark species (e.g., in *Sphyrna lewini* is 1.3%; [15]).

*C. crepidater* exhibits diversities well within the range of other shark species. Values for haplotype diversity range from 0.28 to 0.97 and for nucleotide diversity from 0.0006 to 0.011, in lemon sharks [8] and whale sharks [28] respectively. Interestingly, the closely related Portuguese dogfish *Centroscymnus coelolepis* showed much lower haplotype and nucleotide diversities (0.65 and 0.002, respectively; [6]) than found in *C. crepidater*.

### Historical Divergence in *C. crepidater*

The origin of deep lineages showing no geographic correspondence, such as the two clades found within long-nosed velvet dogfish, can result from two alternative scenarios: vicariance or lineage sorting in a large panmictic population.

#### Vicariance scenario

Bayesian dating analyses indicated an estimate for the divergence of *C. crepidater* into two mitochondrial clades at about 15 myr (Fig. 6). The fossil record of the genus *Centroscymnus* dates back to the Middle Miocene, and the current geographical distribution of the species include both the Atlantic, and Indo-Pacific basins. The Tethys corridor prevailed until ∼17 myr ago with a final closure at 14 myr [14]. Thereby, we first hypothesized that the final closure of the Tethyan corridor could have triggered lineage-splitting events within *C. crepidater*.

Several studies proposed the Tethys vicariant hypothesis to explain the observed divergences between Atlantic and Indo-Pacific lineages (e.g. in lemon [8]; or angel [12]). Although the divergence of the two major *C. crepidater* mitochondrial clades occurred at the end of the closure of this seaway, the predicted outcome of this vicariant episode would be a sister relationship between the eastern Atlantic (one clade) and Indo-West Pacific (another clade) populations. Instead, ML trees (Material S1 and S2) and Bayesian dating analysis (Fig. 6) showed specimens from the Atlantic and Pacific clustering together in one clade whereas the other is predominantly composed of Atlantic samples with a single specimen from the Chatham Rise.

The ML topologies (Material S1 and S2) seem to contradict the proposed hypothesis of divergence of *C. crepidater* driven by the closure of the Tethyan corridor. Nevertheless, this tectonic event could have played an indirect role on species diversification. The closure of this seaway ceased the influx of warm water into the northwestern Indian Ocean causing a significant oceanic cooling [29], [30]. Moreover, the expansion of an Antarctic glaciation during the Middle Miocene (∼17 myr ago) due to the development of a permanent East Antarctic ice sheet affected the Southern Ocean, also contributing to the observed cooling [31], [32].

It is not expected that a marked drop in the deep-water temperature could significantly affect a widespread species occupying a wide range of latitudes such as *C. crepidater*. However, this species feeds mostly on micronektonic organisms (fish and cephalopods) close to the seabed [33], and the oceanic cooling changed the structure of the benthic communities [34]. Fluctuations in the abundance of food resources might have lead to the dispersal of *C. crepidater* throughout the northern Atlantic where habitat conditions were more suitable during the Miocene [35], creating opportunities for lineage isolation. Ultimately, the cooling of the Southern Ocean might have had a vicariant effect by restricting gene flow between basins, which promoted an allopatric divergence of lineages. The well-supported monophyly of the two main lineages of *C. crepidater* (Fig. 6 and Material S1) further support an allopatric origin for the clades.

Two deeply divergent mtDNA clades were described in the bigeye tuna (*Thunnus obesus*) [36], [37], [38] with a spatial genetic structure similar to *C. crepidater*. One bigeye tuna clade was ubiquitously composed of Atlantic and Indo-Pacific samples while a second clade occurred almost entirely in the Atlantic. The lower water temperatures during the peak of glacial periods were proposed to restrict migration between ocean basins promoting divergence [37], [38].

*Centroscymnus coelolepis,* also a benthopelagic shark, does not exhibit any deep genetic break in its Atlantic range [6]. It would be expected that a species sharing with *C. crepidater* an identical geographic distribution and food regime, would be similarly affected by the oceanic cooling observed during the Miocene, and would also display a deep mtDNA divergence. Specimens of *C. crepidater* belonging to the clade II are rare (6.9%), and it is not unlikely that specimens of *C. coelolepis* belonging to the putative second clade have not been sampled.

#### Lineage sorting scenario

The reticulated shape of clade I depicted in the network analysis (Fig. 2) suggests the existence of an old and stable population. Simulations show that large panmictic populations with a stable long history can produce deep mtDNA divergences simply by stochastic lineage sorting [39] as predicted by the coalescence theory [40].

It is difficult to ascertain the origin of a particular phylogeographic outcome because different biogeographical or stochastic events can result in similar population histories [39]. None of the proposed alternatives for the origin of the deep-divergent clades in this species can therefore be discarded. However, the most parsimonious explanation is the allopatric divergence due to a historical vicariant event promoted by the oceanic cooling observed during the Miocene in the Southern Ocean.

### Contemporary Differentiation and Female Philopatry in *C. crepidater*

Microsatellite *C. crepidater* data showed no significant differentiation among Atlantic locations and low levels of gene flow between the Atlantic and Pacific Oceans (Table 2). Estimated inter-oceanic contemporary migration rates were extremely low, in the order of only a few percentages of individuals (less than 4%). Also, estimates indicated that migration is asymmetrical with almost twice the number as many migrants moving from the Pacific towards the Atlantic, than in the opposite direction. Spatial structure revealed the existence of three clusters corresponding to the Atlantic, Pacific, and Great Meteor Bank sample locations (Material S4A). After removing the sample from the Great Meteor Bank (Great Meteor Bank has only 3 scored individuals with high proportion of missing data), the number of clusters included the Pacific and the Atlantic locations only (Material S4B), which is consistent with the existence of present-day barriers to gene flow between these ocean basins.

Nuclear genetic differentiation in marine organisms may have its inception in life-history traits (e.g. poor-dispersal capabilities at any stage of life) or in the presence of oceanic barriers [41]. The lack of a planktonic larval stage in *C. crepidater* implies that dispersal is mostly driven by juvenile or adult mobility and thus, affected to a lesser extent by the action of ocean currents. Benthopelagic species are not expected to exhibit long-range, inter-oceanic active dispersal [1]. However, ocean currents most likely influence the drifting of small fish and crustaceans that are part of the diet of *C. crepidater*.

At the interface between the Atlantic and the Indian Oceans, one of the main oceanographic current is the Agulhas Current (southward flowing) and its retroflection formed by the turning back into the Indian Ocean [42], [43]. Flowing patterns similar to those of today were established only at 5 myr ago [44]. The Agulhas Current retroflection might therefore impact on *C. crepidater* present-day movements by creating a filter to the displacement of its prey between ocean basins. Coastal organisms such as intertidal mussels (*Perna perna*), exhibit distinct lineages around the South Africa coastline, which were attributed to a filter role of the Agulhas Current retroflection [45].

Recent studies on sharks ascribed genetic differentiation to female philopatric behaviour (return to natal sites to breed; [2], [46]). Female philopatry can constitute a reasonable explanation if mtDNA differentiation has a spatial component. Our mtDNA results however, show a deep genetic divergence with no geographic pattern (Table 2) and therefore, do not support the existence of female philopatry in *C. crepidater*. Moreover, results based either in mtDNA or microsatellites showed absence of genetic or geographic structure across putative preferential areas of homing behaviour such as submarine banks (e.g., Anton Dohrn or Rosemary Bank) that are usually used as breeding sites [11].

### Conclusions

Our survey of the deep-sea shark *Centroscymnus crepidater* indicates absence of genetic structure within the Atlantic but significant differentiation between Atlantic and Pacific populations using microsatellite data. Two deeply divergent mtDNA clades were recovered suggesting the occurrence of an allopatric historical event. Bayesian estimates dated this splitting during the Miocene at about 15 myr ago. To a certain extent, the final closure of the Tethys seaway during the Miocene may have been related with the observed lineage split because caused a decrease in water temperature and a subsequent turnover in the oceanic biota. Concomitantly, an Antarctic glaciation also promoted the oceanic cooling. Fluctuations in the food resources, together with the lower temperatures observed in the Southern Ocean during the Miocene, might have lead to the dispersal of *C. crepidater* throughout the northern Atlantic where habitat conditions were more suitable during the Miocene, promoting lineage divergence. We found no evidence of female philopatric behaviour in this species.

## Materials and Methods

### Ethics Statement

All samples from the Azores, Madeira, Mid-Atlantic Ridge (MAR), and Great Meteor Bank (MET) used in this study originated as by-catch from commercial fisheries and were collected by personnel at the Department of Oceanography and Fisheries from the Azores (DOP - http://www.horta.uac.pt/) aboard longliners. Permissions to join the commercial vessel crews were granted by the local Governments of Madeira and Azores for scientific purposes only. MAR and MET sampling locations are located in high seas, which means in legal terms, in areas beyond national jurisdiction, and no further permits are required. In regards of the killing, the majority of deep-sea sharks by the time they reach deck are already dead. For the specimens that were showing signs of life, they were released after a very quick and delicate handling to obtain info on length, weight and sex. Muscle tissue samples were collected from dead specimens.

Samples from Rockall Trough, Anton Dohrn, and Rosemary Bank were collected by research vessels run directly by the UK government through the Annual Survey by Marine Scotland: http://www.scotland.gov.uk/Topics/marine/Sea-Fisheries whose remit specifically entails the monitoring of the status of fish stocks in Scottish waters.

We obtained permits from CITES to import tissue samples from the Tasman Sea through the CSIRO - Marine and Atmospheric Research Institute (Tasmania, Australia). All 22 samples of *Centroscymnus crepidater* specimens from the Tasman Sea used in this study were originated from by-catch collected by Australian commercial fishing vessels using large demersal trawl nets. These vessels have Australian Government permission to conduct fishing operations within Australia's waters. The vessels were either targeting Orange Roughy (*Hoplostethus atlanticus*) or oreo dories (Smooth Oreo - *Pseudocyttus maculatus*, Spikey Oreo - *Neocyttus rhomboidalis* and Black Oreo - *Allocyttus niger*). The sharks would have been brought to surface (from depths of up to 1200 meters) dead amongst the large catch of either Orange Roughy and/or oreo dories. The tissues samples were taken from dead specimens. The specimens were later examined for various biological parameters (sex and development stage, diet and possibly age) by researchers from our divisional shark research team. Samples from the Chatham Rise (southern Pacific Ocean) were obtained through the Bavarian State Collection of Zoology DNA Bank. *Centroscymnus crepidater* is NOT listed in the IUCN list of endangered/vulnerable/threatened species.

### MtDNA and Microsatellites Laboratory Procedures

A total of 132 long-nosed velvet dogfish shark specimens (*Centroscymnus crepidater*) were collected between 2003–2009 from seven locations in the Atlantic Ocean (Table 1). DNA was extracted from muscle tissue stored in absolute ethanol using either a standard salting-out protocol [47] or a phenol-chloroform extraction method followed by ethanol precipitation [48]. Primers ElasmoCR15642 and ElasmoCR16638 developed by [49] were used in polymerase chain reactions (PCRs) to amplify a portion of the mtDNA control region (CR) of *C. crepidater*. Despite using a range of extraction methods and primer combinations, we were only able to amplify 66 out of 132 Atlantic specimens collected using the above-mentioned primers. We amplified DNA of five individuals from the Chatham Rise and of 21 specimens from the Tasman Sea (see Table 1 and Fig. 1 for further details on sample locations). We also amplified a fragment of the cytochrome oxidase subunit I (COI) of a subset of *C. crepidater* samples from the Atlantic and the totality from the Pacific using universal primers from Folmer [50] to further analyse phylogenetic patterns. All phylogenetic analyses were performed for each marker independently, and we included COI for comparison purposes only.

All PCR amplifications were conducted in 25 µl reactions containing 1X PCR buffer (*Buffer BD Advantage 2 PCR* with MgCl_2_), 0.2 mM of each dNTP, 0.2 µM of each primer, 1 µl of template DNA, and Taq DNA polymerase (1 unit, *Taq BD Advantage™ 2 Polymerase Mix;* CLONTECH-Takara). The following program was used for the PCR amplification: one cycle of 1 min at 95°C, 35 cycles of 30 s at 95°C, 30 s at 50°C (COI) or 52°C (CR), and 60 s (COI) or 70 s (CR) at 68°C, and finally, one cycle of 5 min at 68°C. PCR products were purified with an ethanol/sodium acetate precipitation, and directly sequenced using the corresponding PCR primers. Samples were sequenced in an automated DNA sequencer (ABI PRISM 3700) using the BigDye Deoxy Terminator cycle-sequencing kit (Applied Biosystems) following manufacture’s instructions.

Seven species-specific microsatellites (Ccrep02, Ccrep11, Ccrep12, Ccrep13, Ccrep23, Ccrep27 and Ccrep34) multiplexed in a single PCR reaction [27] were employed to analyse the genetic structure of Atlantic and Pacific populations of *C. crepidater* (132 specimens from the Atlantic Ocean and 28 from the Pacific Ocean). PCR amplifications were carried out using Qiagen Multiplex PCR kit in a final volume of 10 µl containing 5 µl of Multiplex Kit Buffer 2× and 2.5 ng/µl of genomic DNA. Final primers’ concentrations and fluorescent labels (Applied Biosystems) were as follows: Ccrep02 [NED] 0.45 µM, Ccrep11 [PET] 0.15 µM, Ccrep12 [6-FAM] 0.3 µM, Ccrep13 [6-FAM] 0.2 µM, Ccrep23 [VIC] 0.25 µM, Ccrep27 [VIC] 0.1 µM and Ccrep34 [NED] 0.15 µM. Amplification started at 95°C for 15 min, followed by 35 cycles of 45 sec at 94°C, 45 sec at 59°C, 45 sec at 72°C, and a final extension step of 45 min at 72°C. PCR products were run alongside a GS600LIZ size standard (Applied Biosystems) in an ABI 3130xl Genetic Analyzer and alleles were scored using the program Genemapper 4.0 (Applied Biosystems).

### Mitochondrial DNA Analyses

CR mtDNA sequences (*C. crepidater*: 66 from the Atlantic Ocean; 21 from the Tasman Sea; five from the Chatham Rise) were aligned with ClustalX [51] using the default settings. In the ML analysis of the CR mtDNA data set, we used five specimens of *Centroscymnus owstonii* (collected from two different locations, Azores and Mid-Atlantic Ridge) and one sequence of *Centroscymnus coelolepis* (retrieved from the GenBank, HQ664449) as an outgroup, given the close phylogenetic relationship between these species [20]. COI mtDNA sequences of *C. crepidater* (24 from the Atlantic Ocean; 22 from the Tasman Sea; six from the Chatham Rise, and sequences available from the GenBank: DQ108233; DQ108234; GU130694) were also aligned with ClustalX. In the ML analysis based on the mtDNA COI data set, we used *Centroscymnus coelolepis* as outgroup (DQ108219).

The Akaike information criterion [52] as implemented in Modeltest v3.7 [53], selected the GTR+Γ (Γ = 0.37) and the HKY+Γ (Γ = 0.19) substitution models that best fit CR and COI data sets, respectively. These settings were used in maximum likelihood (ML) analyses performed with Phyml [54] using CR and COI data sets to assess if the reconstructed phylogenetic patterns using both fragments were congruent. We also used the CR ML tree as starting topology for the dating analysis. The robustness of the inferred trees was tested by nonparametric bootstrapping using 1000 pseudoreplicates.

We used the two-tailed Fisher’s exact test because of the small sample size of clade II, to statistically evaluate the differences in geographical distribution of the two clades.

Haplotype (*h*) and nucleotide (π) diversities [55] were calculated using CR mtDNA sequence data with Arlequin v3.5 [56]. We estimated the level of genetic variation between sample sites using Jost’s *D* [57], which is more appropriate to estimate genetic differentiation between populations as it overcomes some of the limitations of conventional F-statistics [58], [59]. Pairwise *D* and its statistical significance values, obtained by 1000 permutations [60], were estimated using R code. For completeness, we also provide φ_st_ (mtDNA) values generated in Arlequin to readily enable comparison with other studies given the widespread use of this statistic. Genetic diversity within and among populations was estimated with an analysis of molecular variance (AMOVA; [61]) in Arlequin to test for differentiation between the two oceans (Pacific and Atlantic), and within each ocean basin. The significance of all Φ-statistics was tested by 10,000 random permutations of sequences among populations.

Haplotype networks based on CR mtDNA data to depict relationships among haplotypes were performed with Network v4.6 ([62] available at fluxus-engineering.com). Median-joining networks [62] that contained all possible equally short trees were simplified by running the maximum parsimony calculation option to eliminate superfluous nodes and links [63].

Signatures of changes in population size can be detected in the pattern of pairwise differences among mitochondrial sequences [40]. We employed mismatch distributions to visualize signatures of population growth within the two distinct clades of haplotypes identified by the haplotype network. Frequencies of pair sequence differences (mismatch distribution) form a smooth unimodal plot in populations that have undergone recent growth, but not in populations that have had stable sizes for long periods or have declined. Parameters were estimated in Arlequin under the sudden expansion model (null model). The sudden expansion sum of squared deviation (deviation of simulated from observed – SSD) was calculated and tested against that expected under the sudden expansion model. We tested for deviations from neutral expectations using Fu's F_s_ [64] and Ramos-Onsins’ R_2_ [65] statistics. These have been demonstrated to be the most powerful tests available for detecting population growth [65]. Tests of demographic expansion were conducted using DnaSP and significance was evaluated by comparing the observed statistics to a distribution of values generated with 5000 coalescent simulations.

### Microsatellite Analyses: Genetic Diversity and Population Structure

We used genotype and allele frequencies of the microsatellite loci to obtain standard estimates of genetic diversity within and between sample sites. For each microsatellite locus and each location, genetic diversity was assessed with GenoDive v2.0b22 [66] by determining the following parameters: (1) observed number of alleles (A); (2) observed heterozygosity (H_O_); (3) expected heterozygosity (H_E_); (4) population gene diversity (H_S_), and (5) total corrected gene diversity (H’_T_). Allelic richness (A_R_), a sample-size independent measure of the number of alleles, was estimated using Fstat v2.9.3.2 [67]. Genetic structure between and within ocean basins was tested by AMOVA in Arlequin using microsatellite data. We used the StandArich_v1.00 [68], an R package to plot allelic frequencies for each location available at (http://www.ccmar.ualg.pt/maree/software.php?soft=sarich).

We tested for signs of positive and balancing selection using the F_st_-outlier approach [69], [70] implemented in Lositan [71] as a precautionary way to evaluate the possible impact of selection on the genetic structure. Confidence intervals (99%) for neutral loci were determined using 10,000 simulations and also the recommended ‘neutral mean F_ST_’ option [71].

Deviations from the Hardy–Weinberg Equilibrium (HWE) were assessed with the Gis [55] and the Fis statistics from an AMOVA [61] in GenoDive. Differentiation among samples was estimated using Jost’s *D* [57] and calculations were carried out using the R package DEMEtics v. 0.8–5 [72]. The level of significance of Jost’s *D* values was tested using 1,000 bootstraps. For completeness, we also provide F_st_ (microsatellites) values and their statistic significance to readily enable comparison with other studies given the widespread use of this statistic.

Discriminate Analysis of Principle Components (DAPC) [73] as implemented the package Adegenet [74] in R 2.12.1 (R Development Core Team 2010) was performed on multi-locus genotype data for all sampled locations. This method transforms data using principle component analysis (PCA) to create uncorrelated variables for input into Discriminant Analysis (DA). DA maximizes between-group variation and minimizes within-group variation for assessment of population structure. DAPC is free of assumptions about Hardy-Weinberg equilibrium or linkage disequilibrium and provides graphical representation of divergence among populations. The function *find.clust* was employed to investigate genetic structure by running successive *k-*means, using the Bayesian Information Criterion (BIC) and 10^7^ iterations. The optimal *k* is usually associated with the lowest BIC value. In cases of continuously decreasing BIC, the optimal *k* could be selected by visually investigating the rate of decrease [73].

When appropriate, significance levels of all multiple statistical tests were corrected using the false discovery rate (FDR) approach [75], [76] implemented in R using the Qvalue package [77].

Contemporary direction and rate of migration was estimated by a Bayesian method based on multi-locus genotypes implemented in Bayesass [78], by pooling all locations into two groups corresponding to the Atlantic and Pacific Oceans. The method does not assume that populations are at genetic equilibrium or that genotypes are in accord with Hardy-Weinberg equilibrium, but the loci in the parent populations are assumed to be in linkage equilibrium. The method is based on MCMC methods to estimate the posterior probabilities of the migration matrix among sub-populations [78]. Convergence was achieved after 6×10^6^ MCMC iterations and a burn-in of 2×10^6^ steps. The data was run three times to check for consistency of results, and their values were averaged.

Spatial structure was assessed with a Bayesian clustering algorithm to determine the most probable number of genetic clusters present within the dataset without prior knowledge of the individuals’ origin. This method of population assignment was analyzed using the R package Geneland version 2.0 [79] producing a map that consolidated genetic and geographic data. The program makes use of a geographically constrained Bayesian model that explicitly takes into account the spatial position of sampled multilocus genotypes without any prior information on the number of populations and degree of differentiation between them. To determine the number of genetic clusters independent runs were implemented using 1,000,000 MCMC iterations with a burn in period of 100 and a thinning value of 1,000. The value of K was set from 1 to 9 clusters on a correlated frequency model. We inferred the number of clusters from the modal value of K with the highest posterior probability. Geneland is able to detect subtle geographic structure by combining geographic data into the analysis as a weak prior. This follows the assumption that each population exhibits some degree of spatial structure; however, individuals can belong to multiple populations for posterior likelihood testing [79].

### Divergence Time Estimates in *C. crepidater*

Prior to the analysis, identical haplotypes from the mtDNA CR data set were collapsed with the program Collapse version 1.2 [80]. In order to date lineage-splitting events within *C. crepidater*, we used Multidivtime [81], [82] a Bayesian relaxed molecular-clock approach that incorporates variation of rates of evolution among lineages. We followed the procedure outlined in Rutschmann 2005 [83], using the CR ML tree reconstructed in Phyml as a starting phylogeny. Two calibration points were set according to the fossil record. The first calibration was based on the minimum age of the family Somniosidae [19], [84] in which all analysed taxa are included. We imposed an upper bound of 83.5 myr and a lower bound of 70.6 myr (boundaries of the Campanian from the Upper Cretaceous) corresponding to the minimum age of the family. The second calibration was based on the only fossils shark teeth unambiguously assigned to the genus *Centroscymnus* from the Middle Miocene of Japan [21]. We imposed an upper bound of 16.4 myr and a lower bound of 11.2 myr (boundaries of the Middle Miocene) corresponding to the minimum age of the genus. The parameter *rtrate* was set to 0.014 (mean of the prior distribution for the rate at the ingroup root node) and the standard deviation was set to its maximum value (equal to the mean). The MCMC method was employed to approximate both prior and posterior distributions [82] with an initial burn-in period of 100,000 cycles. Markov chain was sampled every 100 cycles until a total of 10,000 samples were collected.

## Supporting Information

Material S1 **Phylogenetic relationships within** ***Centroscymnus crepidater*** **based on a maximum likelihood analysis of an 868 bp fragment of the mitochondrial control region.** Numbers above and below nodes correspond to ML bootstrap proportions and Bayesian posterior probabilities, respectively.
(EPS)

Material S2 **Phylogenetic relationships within** ***Centroscymnus crepidater*** **based on a maximum likelihood analysis of a 655 bp fragment of the mitochondrial cytochrome oxidase subunit I.** Numbers above and below nodes correspond to ML bootstrap proportions and Bayesian posterior probabilities, respectively.
(EPS)

Material S3 **Allele distributions.** Distribution of the alleles found for each locus at the different sites sampled. Note that not all alleles are represented numerically underneath the respective plot. However, the allelic distribution is represented in the graphs for all alleles.
(TIF)

Material S4 **Posterior density distribution of the number of clusters estimated from Geneland analysis.** **A.** Including the Great Meteor Bank sample. **B.** Excluding the Great Meteor Bank sample.
(TIF)
