# Supplementary figures and images for: Ancient Divergence in the Trans-Oceanic Deep-Sea Shark Centroscymnus crepidater
Source: PLoS One. 2012 Nov 8;7(11):e49196. doi: 10.1371/journal.pone.0049196 (PMC3493524; doi:10.1371/journal.pone.0049196)

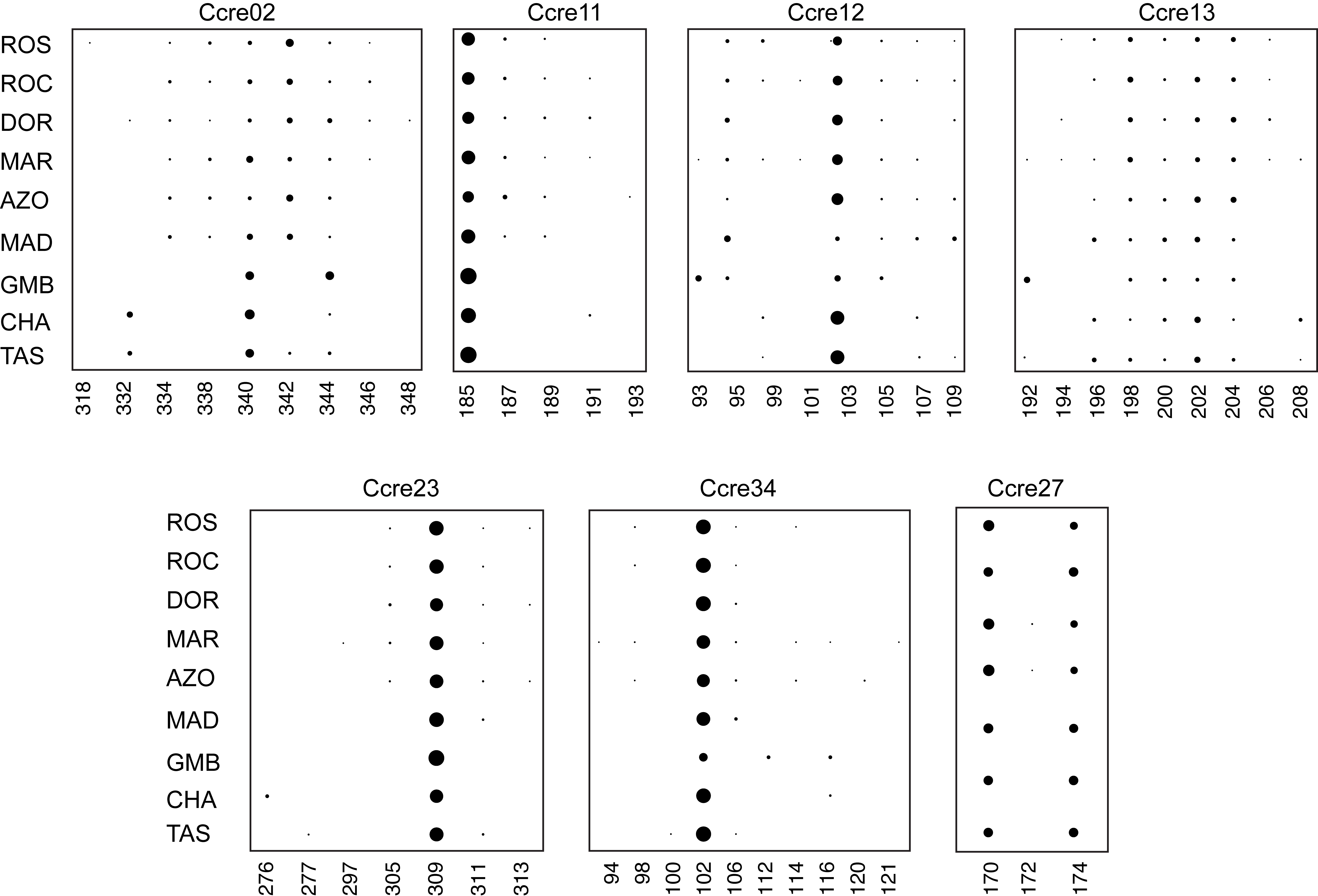

Supplement: Material S3 — Allele distributions. Distribution of the alleles found for each locus at the different sites sampled. Note that not all alleles are represented numerically underneath the respective plot. However, the allelic distribution is represented in the graphs for all alleles. (TIF) [file pone.0049196.s003.tif]

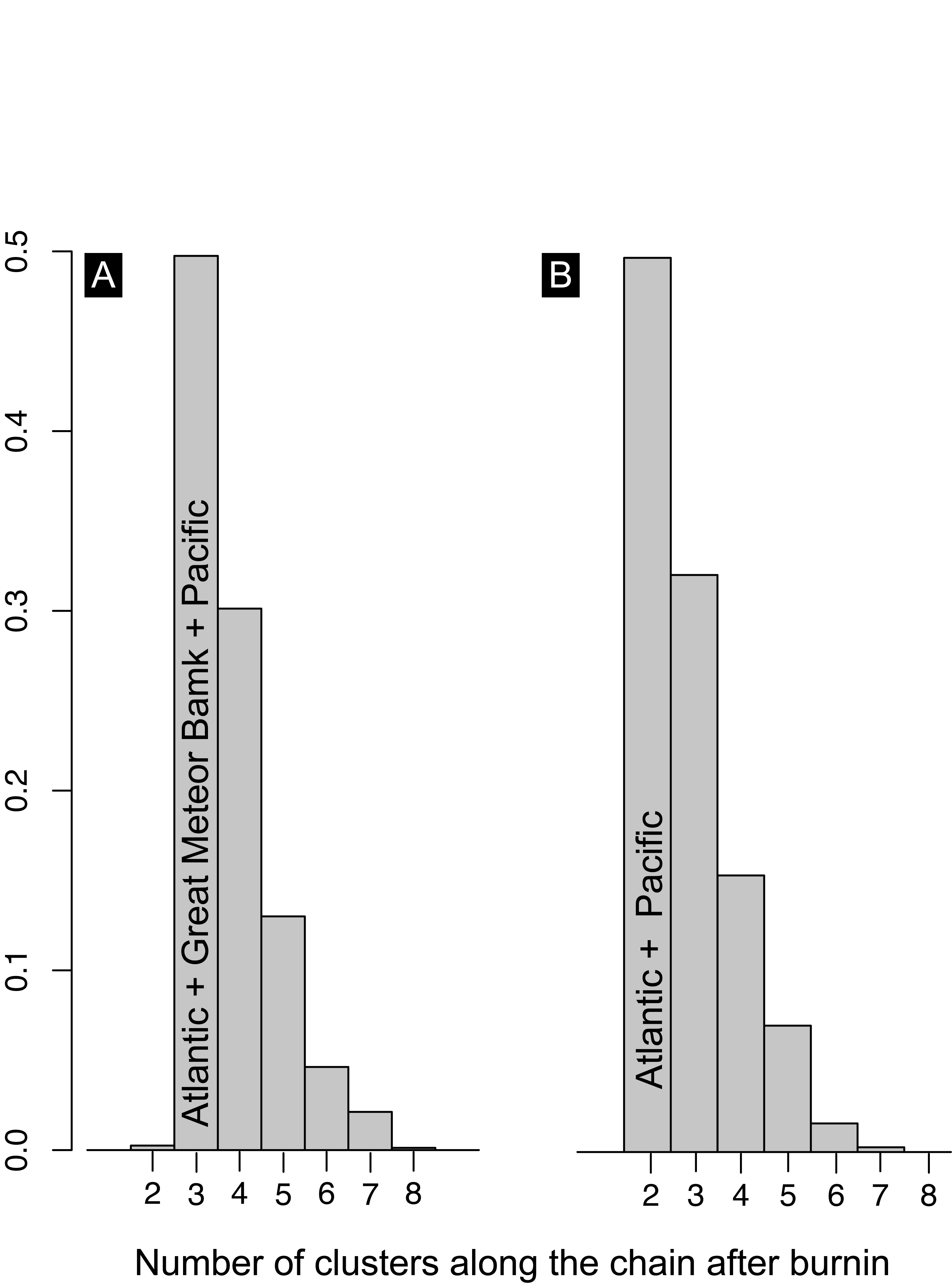

Supplement: Material S4 — Posterior density distribution of the number of clusters estimated from Geneland analysis. A. Including the Great Meteor Bank sample. B. Excluding the Great Meteor Bank sample. (TIF) [file pone.0049196.s004.tif]
